# An OpenSees Surrogate Constitutive Model for High-Damping Rubber Based on Machine Learning

**DOI:** 10.3390/polym16233424

**Published:** 2024-12-05

**Authors:** Feng Li, Tianbo Peng

**Affiliations:** 1 College of Civil Engineering, Tongji University, Shanghai 200092, China; lifeng19@tongji.edu.cn; 2 State Key Laboratory of Disaster Reduction in Civil Engineering, Tongji University, Shanghai 200092, China

**Keywords:** high-damping rubber, machine learning, OpenSees, surrogate constitutive model, finite element analysis

## Abstract

The complex mechanical properties of high-damping rubber (HDR), a commonly used seismic isolation material in buildings and bridges, present a significant challenge in civil engineering. In a previous study, the authors proposed an HDR constitutive model that combines a Gated Recurrent Unit (GRU) and an attention mechanism, offering novel insights into the mechanical properties of HDR. The constitutive model was simplified first to facilitate the deployment of the proposed constitutive model within the finite element analysis environment. Then, the simplified constitutive model was converted into a uniaxial material format suitable for use within the open system for earthquake engineering simulation (OpenSees). In OpenSees, the uniaxial material was named HDRGA material, and the code for the HDRGA material header and source files was written. Finally, an HDR surrogate constitutive model was developed in OpenSees. To validate the precision of the HDRGA material in characterizing the mechanical attributes of HDR, a two-node model and a single-pier model were devised, and their accuracy was verified through a comparative analysis of test results and nonlinear time history calculation results, respectively. The results demonstrate that the developed HDRGA material is capable of performing well under earthquakes.

## 1. Introduction

The rapid improvement of computer processing capabilities has facilitated substantial advancements in the domain of finite element simulation, enabling the analysis of intricate structures with greater precision and efficiency. Nevertheless, the advancement of high-fidelity and accurate simulations remains constrained by the limitations of computational resources and efficiency. The advantage of machine learning technology lies in its capacity to address the issue of scale in numerical solutions. Theoretically, a structure with millions of degrees of freedom can be regarded as a black box, capable of resolving issues that cannot be addressed in real-time due to the constraints of computer performance. The surrogate model based on machine learning algorithms is capable of not only providing the same computational results as finite element algorithms but also significantly improving computational efficiency and reducing computational costs.

In recent years, machine learning has emerged as a prominent research topic in a range of academic disciplines. Nguyen [1] et al. proposed an efficient method based on subset simulation and a surrogate model to study the reliability of building structures under earthquakes. Moustafa [2] et al. utilized Long Short-Term Memory (LSTM) techniques to simulate nonlinear numerical substructures. Guo [3] et al. and Yan [4] et al. put forth a seismic intensity index identifying and evaluation method based on a generalized linear regression model and a Gaussian process regression, respectively. Jia [5] et al. put forth a rapid assessment method for bridge seismic damage based on a random forest algorithm and an artificial neural network. Hwang [6] et al. and Kiani [7] et al. put forth a machine learning-based methodology for the prediction of seismic response. Alwanas [8] et al. employed an extreme learning machine model to predict the bearing capacity and failure mode of concrete in beam–column joints. Lei [9] et al. developed a Bayesian-optimized interpretable ensemble learning surrogate model to predict and interpret the essential seismic requirements of urban highway bridges. Wang [10] et al. employed machine learning algorithms to reliably estimate the bearing deformation and column offset ratio response of bridges.

To accurately and effectively simulate the hysteresis behavior of materials and components, numerous scholars have conducted extensive research based on machine learning.

Regarding the hysteresis behavior of seismic isolation devices, Zhang [11] et al. conducted static cyclic tests on laminated rubber bearings, taking into account the initial stiffness, friction coefficient, cross-sectional area, height, loading speed, vertical load, and aging time of the bearings. A novel constitutive model for bearings was devised through the application of artificial neural network technology. An artificial neural network-based bridge earthquake demand model was developed and implemented for the expeditious evaluation of bridge damage. The findings demonstrate that the artificial neural network earthquake demand model is capable of accurately fitting the complex functional relationship between a multitude of factors and bridge seismic response, thereby facilitating a rapid assessment of bridge seismic damage. Nasab [12,13] et al. developed a kriging model to address the issue of traditional modeling methods being unable to accurately simulate the mechanical properties of viscoelastic dampers that vary with different loading conditions, including input frequency, amplitude, and temperature. The model was utilized for the fragility analysis of structures reinforced with viscoelastic dampers. The findings demonstrate that the model exhibits high accuracy in forecasting experimental outcomes and is capable of effectively simulating the seismic performance of viscoelastic dampers with uncertain parameters. Mekaoui [14,15] et al. put forth a hybrid seismic analysis method for calculating the fully nonlinear response of building structures, which they validated using deep learning. The objective is to predict the nonlinear hysteresis response of seismic isolation devices with deformation and velocity-related characteristics. The results demonstrate that the integration of mechanics-based and data-driven methodologies through a hybrid analysis approach represents an effective methodology for simulating the response of buildings.

Regarding the hysteresis behavior of structural components and materials, Ni [16] et al. employed an enhanced bidirectional LSTM and bidirectional Gated Recurrent Unit (GRU) to assess the hysteresis behavior of HRB600 reinforced concrete columns subjected to repeated loading. Their analytical capabilities were then compared with those of finite element analysis. The findings demonstrate that the selected methodology is effective in simulating hysteresis curves. However, the GRU approach demonstrates the most precise predictive capability, as evidenced by the highest R^2^ and lowest MAE, MSE, and RMSE values. Xu [17] et al. put forth a deep learning-based predictive simulation framework that offers precise and expedient hysteresis models for structural analysis. The analysis of the improved steel support model and the Bouc–Wen hysteresis model demonstrates that the model is both highly accurate and computationally efficient. Gu [18] et al. put forth a sensitivity-guided LSTM neural network methodology for the precise and expedient extraction of structural behavior features and the prediction of pivotal parameters associated with explicit hysteresis models. The findings demonstrate that this approach can accurately predict structural behavior, is more effective than manual construction, and exhibits superior generalization ability compared to classical data-driven hysteresis models. Xu [19] et al. constructed a deep neural network model comprising four hidden layers and one hundred units per layer to predict the complete residual stress–strain response of ultra-high performance concrete materials after exposure to high temperatures. The findings demonstrate that the stress–strain curve predicted by the deep neural network model exhibits a high degree of consistency with the UHPC test curve. The above studies show that the existing research has not yet integrated machine learning-based hysteresis behavior studies with finite element calculations.

High-damping rubber (HDR) bearings have been extensively utilized in the seismic design of building and bridge engineering. The mechanical properties of HDR are primarily influenced by temperature and loading cases. In the field of constitutive model research for HDR, several scholars have established constitutive models based on different theoretical frameworks.

Although hyperelastic constitutive models have been studied for nearly eighty years, choosing a model that accurately describes the mechanical response of rubber remains a challenge. He [20] et al. developed a fitting algorithm to quantitatively evaluate the ability of each strain energy function to reproduce experimental data of unfilled and highly filled rubber nanocomposites by reviewing eighty-five isotropic strain energy functions based on phenomenological theory and micromechanical network theory proposed from the 1940s to 2019. Xiang [21] et al. reviewed the constitutive models of physics-based soft materials to explain hyperelasticity, viscoelasticity, and damage phenomena. The quantitative comparison of hyperelastic models guides the selection of appropriate constitutive models.

Arruda [22] et al. proposed a constitutive model for the deformation of rubber materials based on the eight-chain representation of the underlying macromolecular network structure of rubber and the non-Gaussian behavior of individual chains in the proposed network. The model successfully represented the responses of these materials in uniaxial tension, biaxial tension, uniaxial compression, plane strain compression, and pure shear. Ogden [23] et al. believed that the common practice of writing strain energy as a function of two independent strain invariants often complicates the related mathematical analysis. Therefore, by fully utilizing the inherent simplicity of rubber’s isotropic elasticity, a strain energy function was constructed. This function (i) provides a sufficient representation of the mechanical response of rubbery solids, and (ii) is simple enough for mathematical analysis. Han [24] et al. studied the complete constitutive relationship of hyperelastic materials within the theoretical framework of continuum mechanics, starting from the strain energy function. The research results indicate that based on experimental curves of the full deformation range under multiple deformation modes, the complete constitutive relationship of hyperelastic materials can be obtained, which has guiding significance for theoretical research and engineering applications of complex practical problems such as the fracture of hyperelastic materials. Wei [25] et al. conducted multi-step relaxation tests and cyclic shear tests under different compression forces and loading rates to investigate the effects of loading rates and compression forces on the mechanical behavior of HDR bearing. An HDR-bearing rate-dependent constitutive model considering the influence of compressive force was proposed, and its effect on structural seismic response was studied. Wei [26] et al. proposed a new elastic incompressible isotropic constitutive model for rubber-like materials. This model is an isotropic incompressible model defined based on the first and second principal stretches and is applicable to describe the elasticity of rubber-like materials in general deformation states. Wang [27] et al. proposed a multi-axial compressible strain energy function and directly and explicitly simulated the stress–strain hysteresis loop generated by the Mullins effect in rubber-like materials during loading and unloading cycles. Reese [28] et al. proposed a finite deformation viscoelastic model that employs nonlinear evolution laws and compared it with other models. Ghosh [29] et al. provided two potential frameworks for the constitutive modeling of dielectric elastomers, considering deformation-enhanced shear thinning due to viscosity dissipation.

It shows that the majority of existing studies have concentrated on monotonic loading test data under tension or compression. The findings of this study indicate that the strain history of HDR has a significant impact on its mechanical properties. It is a challenging task to establish a constitutive model that can accurately simulate the influence of various main factors on HDR. Furthermore, a comprehensive consideration of the Mullins effect represents a significant challenge. Accordingly, in the authors’ previous research [30], an HDR constitutive model combining a GRU and an attention mechanism was proposed.

In this study, we initially present the HDR constitutive model, which combines a GRU and an attention mechanism. We then proceed to further simplify this constitutive model. Subsequently, the simplified constitutive model is transformed into a new open system for earthquake engineering simulation (OpenSees) uniaxial material HDRGA through format conversion. This enables the accurate description of the force–displacement relationship of HDR bearings when they are incorporated into finite element calculations.

## 2. Materials and Methods

### 2.1. Test Specimen and Cases

In engineering applications, HDR bearing is primarily responsible for bearing the vertical force transmitted by the upper structure and the horizontal force under earthquakes. Accordingly, this study employed multi-case compression shear testing on HDR specimens.

**Figure 1:**
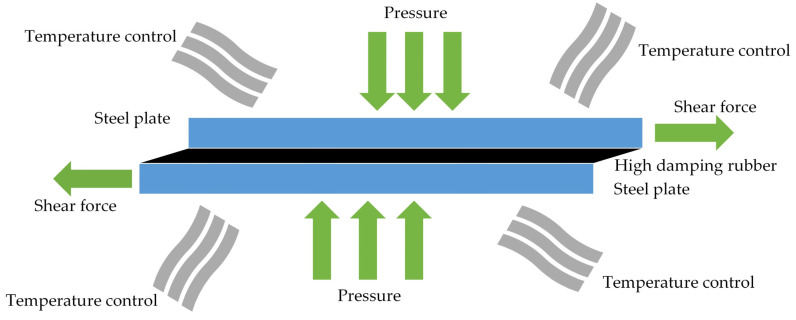
The HDR specimen.

The HDR specimen is composed of two steel plates measuring 250 mm × 250 mm × 20 mm, with a single HDR layer measuring 250 mm × 250 mm × 5 mm sandwiched between them. The steel plates and HDR layer are bonded together through vulcanization, as illustrated in Figure 1. For temperature control, the high and low temperature alternating environment test chamber is used. The 100 t electro-hydraulic servo actuator is used as the horizontal loading equipment, while the 200 t vertical actuator is used as the vertical loading equipment. The cases for HDR specimens are shown in Table 1. Among them, the training cases include five temperatures and five strain rates. The testing cases include four temperatures and one strain rate. Each test case undergoes two types of strain amplitude loading processes: amplitude-increasing cyclic loading and amplitude-decreasing cyclic loading.

### 2.2. LSTM and GRU

HDR has complex nonlinear constitutive relationships. However, explicit formulas are typically constrained by limited parameters, and constitutive models established through explicit formulas ultimately suffer from inaccurate descriptions. The constitutive model of HDR exhibits evident time-series characteristics, necessitating comprehensive investigation through techniques capable of integrating previous information, which has a profound impact on the current state.

A recurrent neural network (RNN) is a special type of large model neural network that is particularly suitable for processing and predicting temporal dependencies and temporal information in sequential data. Unlike traditional feedforward neural networks, an RNN has connected nodes between hidden layers, allowing them to store and transmit information from previous time steps to the current time step. An RNN generally takes sequence data as input and effectively captures the relationship features between sequences through the internal structure design of the network. It is usually output in the form of sequences. The recurrent mechanism of an RNN enables the results generated in the previous time step of the model’s hidden layer to be used as part of the input for the current time step (the input for the current time step includes not only the normal input but also the output of the previous hidden layer), which affects the output of the current time step. However, an RNN has not been able to address the issue of long-term dependencies [31].

The LSTM [32] is a special type of RNN. LSTM aims to solve the problem of gradient vanishing or exploding encountered by traditional RNNs when processing long sequence data. Compared to standard RNNs, LSTM introduces more complex structures to maintain and update internal states. The GRU is a simplified adaptive approach derived from the LSTM. It has been demonstrated to achieve performance comparable to that of the LSTM [33].

**Figure 2:**
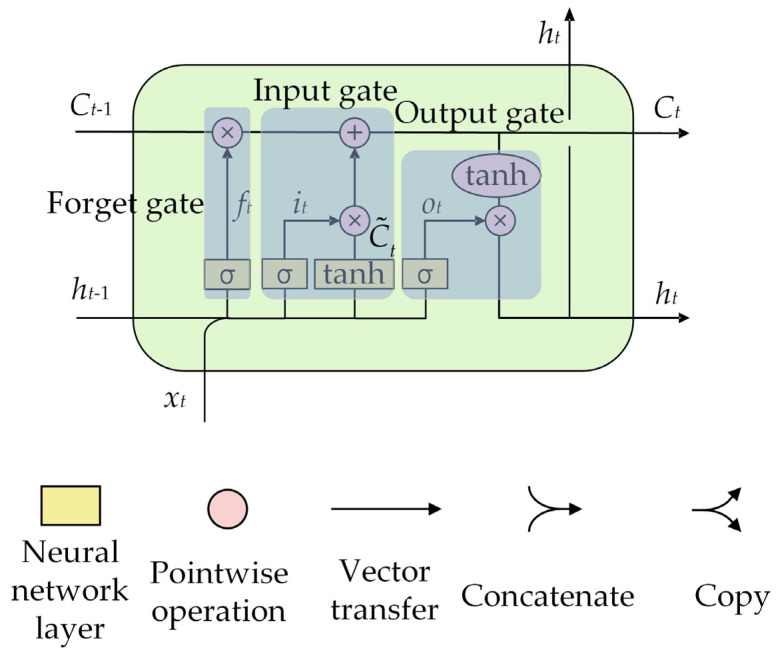
The LSTM structure.

The components of the LSTM [34] are shown in Figure 2. The yellow box represents the learning neural network layer comprising the σ network layer and the tanh network layer. The activation functions associated with these layers are the Sigmoid and tanh functions, represented by Equations (1) and (2), respectively. The pink circle signifies the point-by-point operation, denoting vector multiplication as “⊗” and vector addition as “⊕”. Each line carries an entire vector from the output of one node to the inputs of others. The merging of lines indicates the concatenation of information, while the bifurcated line signifies the copy of the information, which is subsequently passed to the corresponding location. These components form the input gate, forget gate, and output gate, with the respective calculation equations outlined in Equations (3)–(8).

(1) $$\mathrm{Sigmoid}(x)=\frac{1}{1+e^{-x}}$$

(2) $$\tanh(x)=\frac{e^{x}-e^{-x}}{e^{x}+e^{-x}}$$

(3) $$f_{t}=\mathrm{Sigmoid}\left(W_{f}\cdot \left[h_{t-1},x_{t}\right]+b_{f}\right)$$

(4) $$i_{t}=\mathrm{Sigmoid}\left(W_{i}\cdot \left[h_{t-1},x_{t}\right]+b_{i}\right)$$

(5) $${\tilde{C}}_{t}=\tanh\left(W_{C}\cdot \left[h_{t-1},x_{t}\right]+b_{C}\right)$$

(6) $$C_{t}=f_{t}\times C_{t-1}+i_{t}\times {\tilde{C}}_{t}$$

(7) $$o_{t}=\mathrm{Sigmoid}\left(W_{o}\cdot \left[h_{t-1},x_{t}\right]+b_{o}\right)$$

(8) $$h_{t}=o_{t}\times \tanh\left(C_{t}\right)$$

where *f_t_*, *i_t_*, and *o_t_* are the activation values of the forget gate, input gate, and output gate, respectively; ${\tilde{C}}_{t}$ and *C_t_* are the candidate memory unit state and updated memory unit state, respectively; *h_t_* is the final hidden state output; *W_f_*, *W_i_*, *W_C_*, and *W_o_* are the weight matrix, respectively; *b_f_*, *b_i_*, *b_C_*, and *b_o_* are the bias term, respectively; *h_t_*_−1_ and *x_t_* are the hidden state of the previous time step and the input of the current time step, respectively; *C_t_*_−1_ is updated memory unit state of the previous time step.

**Figure 3:**
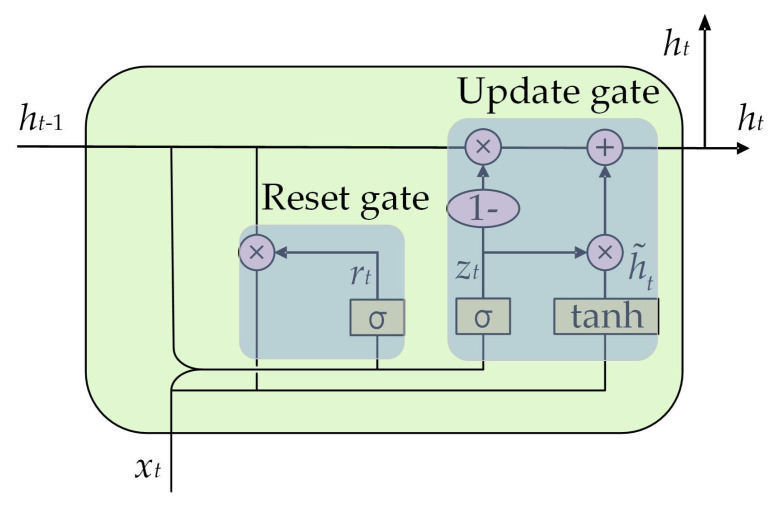
The GRU structure.

Building upon the LSTM architecture, the GRU simplifies the gating mechanism by condensing the input gate, forget gate, and output gate of LSTM into a reset gate and an update gate. The structure of GRU [34] is illustrated in Figure 3. Each component is identical to its LSTM counterpart, and the corresponding calculation equations are presented as Equations (9)–(12). In comparison to LSTM, GRU exhibits a reduction in the number of parameters, making it more manageable for training and tuning while also enhancing computational efficiency.

(9) $$z_{t}=\mathrm{Sigmoid}\left(W_{z}\cdot \left[h_{t-1},x_{t}\right]\right)$$

(10) $$r_{t}=\mathrm{Sigmoid}\left(W_{r}\cdot \left[h_{t-1},x_{t}\right]\right)$$

(11) $${\tilde{h}}_{t}=\tanh\left(W\cdot \left[r_{t}\times h_{t-1},x_{t}\right]\right)$$

(12) $$h_{t}=\left(1-z_{t}\right)\times h_{t-1}+z_{t}\times {\tilde{h}}_{t}$$

where *z_t_* and *r_t_* are the activation values of the update gate and reset gate, respectively; ${\tilde{h}}_{t}$ is the candidate memory unit state; *h_t_* is the final hidden state output; *W_z_*, *W_r_*, and *W* are the weight matrix, respectively; *h_t_*_−1_ and *x_t_* are the hidden state of the previous time step and the input of the current time step, respectively.

### 2.3. GRU + Attention Model Simplification

The attention mechanism is a commonly used technique in deep learning that plays a crucial role in effectively using input information by applying different weights based on specific requirements [35]. By incorporating the attention mechanism into the GRU model, it becomes possible to fully explore the distinctive characteristics of data and use the most significant components within the time series data for modeling purposes. This integration enhances the model performance by emphasizing relevant information. By incorporating the attention mechanism, the GRU model becomes capable of selectively attending to critical features, improving its ability to capture complex characteristics and enhance prediction accuracy.

Details regarding the specific model structure and prediction performance can be found in reference [30].

**Figure 4:**
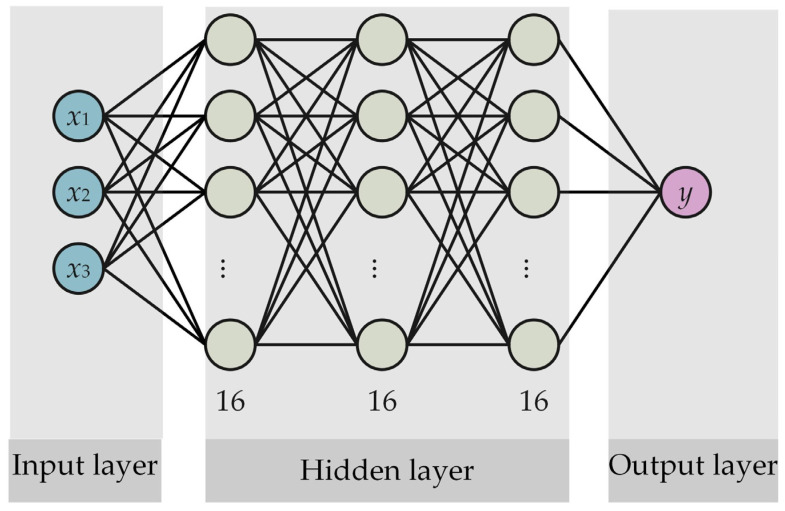
The MLP structure.

The primary objective of model simplification is to streamline the processing of data with a length that is less than the sliding window length. The multi-layer perceptron (MLP) is one of the most basic neural network models, with a relatively simple structure that is easy to understand and implement. It also has good scalability and universality and can be applied to various tasks such as classification and regression. In reference [30], an MLP is employed for training and prediction concerning this particular subset of the data. The MLP structure comprises three hidden layers, each comprising 16 units, as illustrated in Figure 4.

While the aforementioned method can also yield satisfactory prediction outcomes, it is necessary to employ an MLP to generate data of an equivalent length to the sliding window before utilizing the GRU + attention model for prediction. From an examination of the overall model structure and prediction process, it can be seen that the method is somewhat cumbersome.

Accordingly, in the present study, the MLP component will be eliminated, and the prediction issue when the data length is insufficient for the sliding window length will be addressed by appending “0” to the test data. The specific approach is to insert an equal number of “0”s as the sliding window length at the beginning of each training case data, thereby enabling the GRU + attention model to be trained on a sequence comprising solely “0”s.

### 2.4. OpenSees Uniaxial Material Development

OpenSees is an open-source software framework [36] that is primarily utilized for the simulation and analysis of earthquake engineering and structural systems. The software includes a comprehensive material library, offering a variety of material models to address diverse engineering requirements. The models encompass a range of behaviors, from simple elastic behavior to complex nonlinear and damage behavior, and are suitable for a variety of structural simulations. Additionally, OpenSees enables users to develop their material models tailored to specific requirements, offering considerable flexibility.

The existing uniaxial material used to describe the constitutive relationship of HDR in OpenSees is KikuchiAikenHDR material. However, this material is unable to account for the influence of external factors on the constitutive relationship of HDR or to incorporate the Mullins effect of HDR.

#### 2.4.1. KikuchiAikenHDR Material

The KikuchiAikenHDR material in OpenSees uniaxial material is used to simulate the nonlinear hysteresis behavior of HDR, and its material command is as follows:

uniaxialMaterial KikuchiAikenHDR $*matTag* $*tp* $*ar* $*hr*

where $*matTag* is the material number, $*tp* is the HDR type, $*ar* is the HDR area, and $*hr* is the total HDR thickness.

From the material command, it can be seen that the KikuchiAikenHDR material can only be used for simulating the hysteresis behavior of HDR simplification. It is not capable of considering the effects of temperature, loading rate, and other factors, nor can it describe the Mullins effect of HDR.

To address the shortcomings of the KikuchiAikenHDR material and to capitalize on the benefits of the simplified GRU + attention model, an HDR OpenSees surrogate constitutive model was developed. The surrogate constitutive model was named HDRGA material.

#### 2.4.2. HDRGA Material

**Figure 5:**
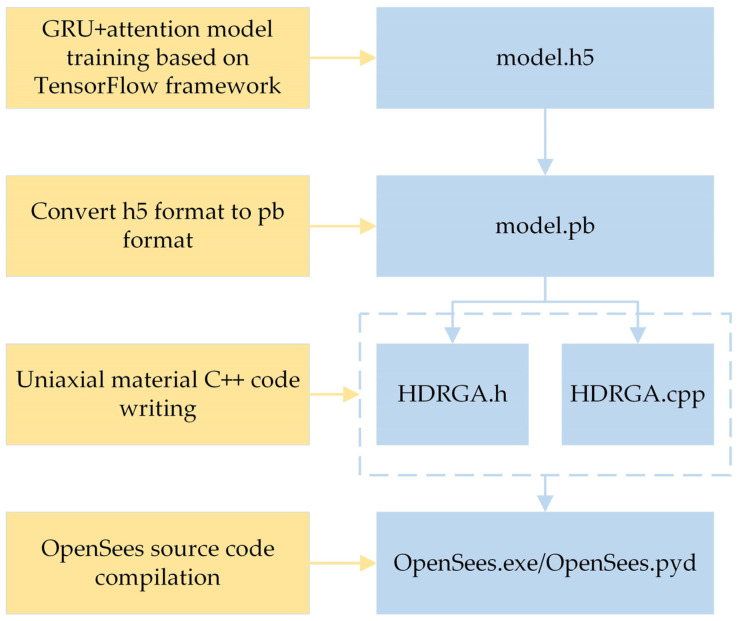
The HDRGA material development process.

The HDRGA material has been developed based on the latest version of the OpenSees open-source code, version 3.6.0. As OpenSees is primarily developed in the C++ language, machine learning models trained in Python 3.9 cannot be directly applied within OpenSees. Accordingly, the initial step is to train the GRU + attention model, simplified as detailed in Section 2.3, in Python to generate a “model.h5” file. This is then converted to a “model. pb” file, and, finally, C++ code is written within the OpenSees. The specific development process is illustrated in Figure 5. The final result is OpenSees.exe, which can be directly executed for structural calculations or to replace the file with the same name in OpenSeespy with OpenSees.pyd. Subsequently, structural calculations can be performed through Python calls.

In OpenSees, the command for accessing the HDRGA material is as follows:

uniaxialMaterial HDRGA $*matTag* $*nnid* $*temp*

where $*matTag* is the material number, $*nnid* is the neural network number, and $*temp* is the ambient temperature.

Next, the accuracy of the HDRGA material will be verified.

## 3. Results and Discussion

### 3.1. Test Results Verification

**Figure 6:**
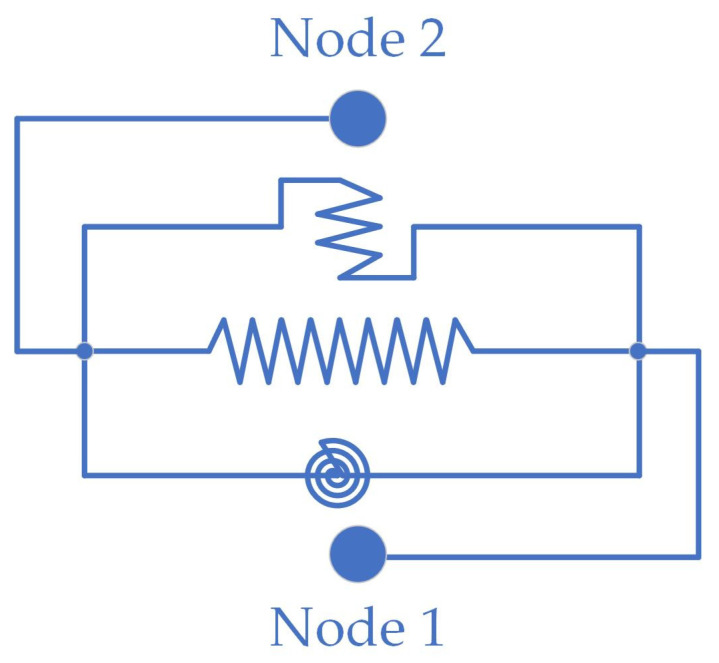
The two-node model.

**Figure 7:**
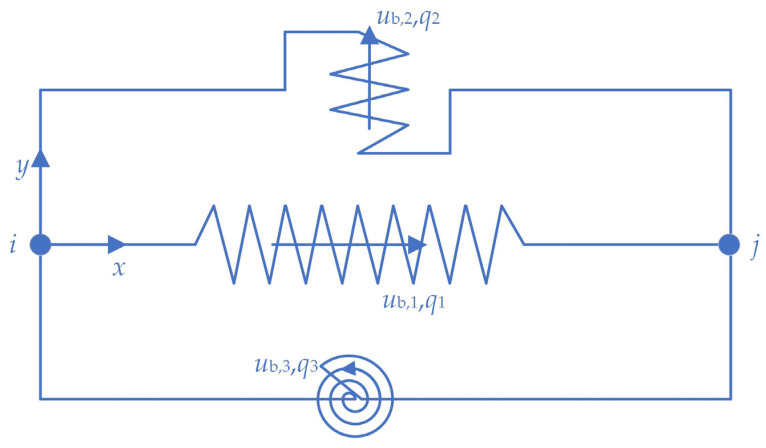
The two-node link element [36]. Where *x*, *y* are the local coordinate axes of the element, *i*, *j* are the nodes at both ends of the element, and *u*_b,*i*_, *q_i_* are the displacement and force in the corresponding direction of the element, respectively.

To verify the accuracy of the description of the HDR mechanical properties provided by the HDRGA material, a two-node model was constructed in OpenSees. Node 1 was designated as a fixed node, while node 2 was configured to release one degree of freedom in a single direction, as illustrated in Figure 6. The two nodes are connected by a two-node link element, which is assigned the HDRGA material type, as illustrated in Figure 7. Subsequently, the time history of shear displacement corresponding to the specified test cases is applied to node 2 through displacement loading to verify whether the shear force generated by the HDRGA material is in alignment with the test results.

**Figure 8:**
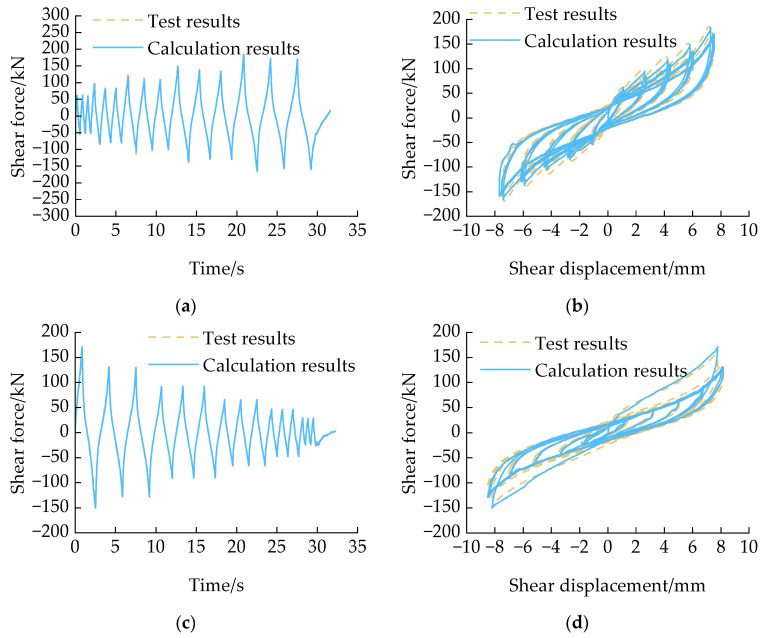
The verification results. (**a**) Shear force time history of amplitude-increasing loading at a temperature of 260.65 K; (**b**) shear force–displacement relationship of amplitude-increasing loading at a temperature of 260.65 K; (**c**) shear force time history of amplitude-decreasing loading at a temperature of 305.65 K; (**d**) shear force–displacement relationship of amplitude-decreasing loading at a temperature of 305.65 K.

The accuracy of the HDRGA material was demonstrated by taking the temperature of 260.65 K amplitude-increasing loading and 305.65 K amplitude-decreasing loading as examples in the testing cases. The specific comparison results are shown in Figure 8. As illustrated in Figure 8, the shear force results calculated through OpenSees are found to be essentially consistent with the test results. Furthermore, the GRU+attention model is demonstrated to be capable of accurately describing the shear force–displacement relationship under both amplitude-increasing loading and amplitude-decreasing loading. This demonstrated the feasibility of introducing the GRU + attention model into OpenSees.

### 3.2. Nonlinear Time History Verification

**Figure 9:**
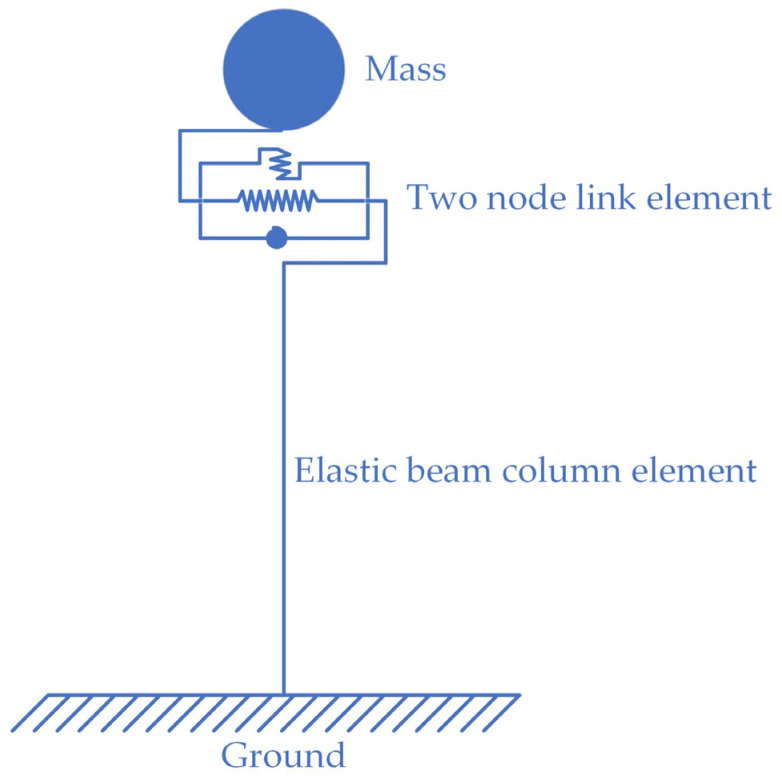
The single-pier model.

**Figure 10:**
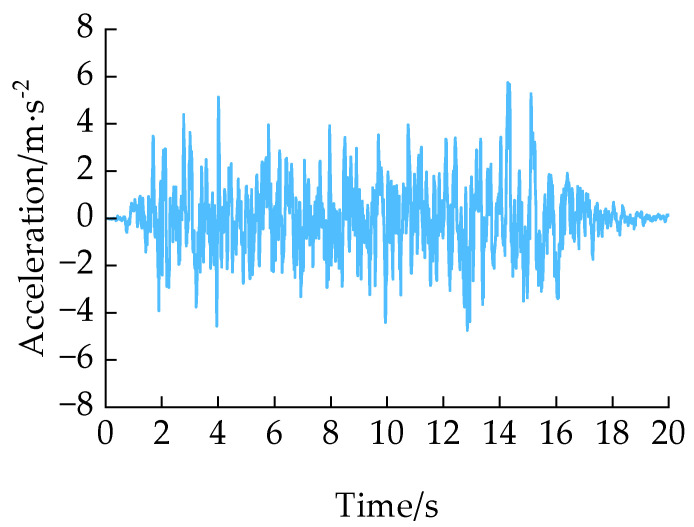
The artificial seismic wave.

To ascertain whether the HDRGA material is capable of functioning correctly in the event of an earthquake, a single-pier model was constructed and simulated using an elastic beam column element. The lumped mass at the top of the pier is connected by a two-node link element, which is assigned the HDRGA material type, as illustrated in Figure 9. The lumped mass is 518.4 t, the pier body has an equal cross-section with a cross-sectional area of 17.05 m^2^, a pier height of 5 m, an elastic modulus of 3.15 × 10^10^ Pa, a cross-sectional moment of inertia of 11.27 m^4^, and the mass per unit length of the pier body is 40.75 t/m. In this structure, the HDRGA material corresponds to an HDR bearing area of 720 mm × 720 mm, a total HDR thickness of 140 mm, and a temperature of 313.15 K. An artificial seismic wave was generated using SeismoSignal software 2024, as illustrated in Figure 10. The peak acceleration of the seismic wave is 5.74 m/s^2^, with a duration of 20 s.

**Figure 11:**
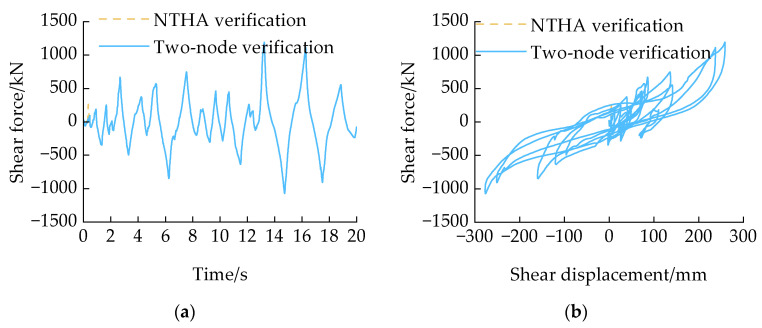
The nonlinear time history verification results. (**a**) Shear force time history; (**b**) shear force–displacement relationship.

The hysteresis curve of the HDRGA material under seismic wave action was calculated through nonlinear time history analysis (NTHA), as illustrated by the black dashed line in Figure 11. To ascertain the extent to which the constitutive behavior of the HDRGA material accurately reflects the results of the NTHA, the shear displacement of the HDRGA material obtained from the NTHA was applied to node 2 in Figure 6. The corresponding hysteresis curve was calculated and is shown by the red solid line in Figure 11. As illustrated in Figure 11, the hysteresis curve calculated through NTHA is in complete alignment with the hysteresis curve validated by the two-node model. This demonstrates that the HDRGA material is fully capable of meeting the structural calculation requirements under earthquakes.

## 4. Conclusions

An OpenSees surrogate constitutive model for HDR based on machine learning was developed by simplifying the GRU + attention constitutive model and deploying it to OpenSees. The following conclusions were drawn:

(1) The constitutive relationship of HDR materials has complex nonlinear characteristics, and existing HDR constitutive models make it difficult to fully describe the influence of multiple factors on their stress–strain relationship. The GRU + attention approach can effectively solve the problems in existing HDR constitutive models.

(2) The development of a material surrogate constitutive model has the potential to markedly reduce the consumption of computational resources, considerably enhance computational efficiency, and reduce computational costs while ensuring the expeditious and precise simulation of material constitutive relationships.

(3) The development of HDRGA material provides a novel approach for expediently comprehending materials with complex properties and implementing them in finite element calculations.

## Figures and Tables

**Table 1:** The cases for the HDR specimen.

Case	Temperature/K	Strain Rate/s^−1^	Strain Amplitude/%
Training case	253.15	0.2	40 → 80 → 120 → 160 → 200 and 200 → 160 → 120 → 80 → 40
	253.15	0.4	
	253.15	0.8	
	253.15	1.6	
	253.15	3.2	
	268.15	0.2	
	268.15	0.4	
	268.15	0.8	
	268.15	1.6	
	268.15	3.2	
	283.15	0.2	
	283.15	0.4	
	283.15	0.8	
	283.15	1.6	
	283.15	3.2	
	298.15	0.2	
	298.15	0.4	
	298.15	0.8	
	298.15	1.6	
	298.15	3.2	
	313.15	0.2	
	313.15	0.4	
	313.15	0.8	
	313.15	1.6	
	313.15	3.2	
Testing case	260.65	2.4	
	275.65	2.4	
	290.65	2.4	
	305.65	2.4	

## Data Availability

The raw data supporting the conclusions of this article will be made available by the authors on request.
